# Racotumomab: an anti-idiotype vaccine related to *N*-glycolyl-containing gangliosides – preclinical and clinical data

**DOI:** 10.3389/fonc.2012.00150

**Published:** 2012-10-23

**Authors:** Ana M. Vázquez, Ana M. Hernández, Amparo Macías, Enrique Montero, Daniel E. Gómez, Daniel F. Alonso, Mariano R. Gabri, Roberto E. Gómez

**Affiliations:** ^1^Center of Molecular ImmunologyHavana, Cuba; ^2^Quilmes National UniversityBuenos Aires, Argentina; ^3^ELEA LaboratoriesBuenos Aires, Argentina

**Keywords:** anti-idiotype vaccine, cancer, immunotherapy

## Abstract

Neu-glycolyl (NeuGc)-containing gangliosides are attractive targets for immunotherapy with anti-idiotype mAbs, because these glycolipids are not normal components of the cytoplasmic membrane in humans, but their expression has been demonstrated in several human malignant tumors. Racotumomab is an anti-idiotype mAb specific to P3 mAb, an antibody which reacts to NeuGc-containing gangliosides, sulfatides, and other antigens expressed in tumors. Preparations containing racotumomab were able to induce a strong anti-metastatic effect in tumor-bearing mice. Different Phase I clinical trials have been conducted in patients with advanced melanoma, breast cancer, and lung cancer. The results of these clinical trials demonstrated the low toxicity and the high immunogenicity of this vaccine. The induced antibodies recognized and directly killed tumor cells expressing NeuGcGM3. A Phase II/III multicenter, controlled, randomized, double blind clinical trial was conducted to evaluate the effect of aluminum hydroxide-precipitated racotumomab vaccine in overall survival in patients with advanced non-small cell lung cancer. The clinical results of this study showed a significant clinical benefit in the patients who were treated with the anti-idiotype vaccine.

Actively induced immunotherapy is one of the most promising fields in cancer research and numerous approaches are being studied to design effective cancer vaccines. An approach to generate an effective immune response against tumor-associated antigens involves the use of an anti-idiotype monoclonal antibody (Ab2 mAb). The use of Ab2 as a vaccine is a consequence of Jerne’s idiotypic network theory, which postulates that, due to the large potential diversity of immunoglobulin variable regions, the idiotype repertoire can mimic the universe of self and foreign epitopes (Jerne, 1974). Thus, properly selected anti-idiotypic antibodies could act as tumor-associated antigen surrogates.

Studies in animal models have demonstrated the efficacy of these vaccines for triggering the immune system to induce specific and protective immunity against tumors of different origin (Bhattacharya-Chatterjee et al., 2001; Mohanty et al., 2007). In the clinical setting anti-idiotypic antibodies are also being actively evaluated for the treatment of malignant diseases (de Cerio et al., 2007). In fact, several studies have validated the capacity of anti-idiotype mAbs to mimic different kind of antigens in cancer patients, inducing humoral and cellular responses against the nominal antigens. One of these antibodies designated 3H1 mimics a specific epitope on the carcinoembryonic antigen (CEA), a tumor-associated antigen expressed on most gastrointestinal adenocarcinomas. This anti-idiotype mAb was used in Phase I clinical trials in advanced colorectal cancer patients and demonstrated specific active immunity to CEA, inducing anti-CEA antibodies and T cell proliferative responses. The level of the immune response correlated directly with the time to progression and survival. More recently these researchers performed a Phase III clinical trial with 630 patients with advanced colorectal cancer combining 5-fluorouracil and leucovorin with either 3H1 anti-idiotype mAb or placebo. Although the addition of 3H1 did not improve the overall patient outcome, again there was an improvement in the survival of the patients that developed strong anti-CEA responses (Foon et al., 1995, 1997, 1999; Bhattacharya-Chatterjee et al., 2000; Chong et al., 2006).

Mittelman et al. (1990), 1992 demonstrated the capacity of the anti-idiotype mAb MK2-23 to mimic a high molecular weight human melanoma antigen (HMW-MAA) and to induce an anti-tumor response in melanoma patients, which included the reduction in the size of metastatic lesions in some of the patients. In these studies the survival of the patients who developed anti-HMW-MAA antibodies was also significantly longer.

An anti-idiotype antibody that has shown to activate T cell responses against the nominal antigen is the 105AD7. This antibody has both amino acid and structural homology with the complement regulatory protein CD55, a glycoprotein that protects the cells from the attack by complement and which is overexpressed in osteosarcoma and colorectal cancer cells. Some of the osteosarcoma patients vaccinated with 105AD7 elicited an IFN-gamma T cell response against the vaccine, and TNF-alpha and GM-CSF responses not only to the vaccine, but also toward the native antigen. This antibody was also injected in colorectal cancer patients, where again it induced proliferative responses and TNF-alpha and GM-CSF secretion against the nominal antigen. These researchers have not seen correlation between the induced T cell responses and patient’s survival, probably due to the small number of patients included in the studies (Robins et al., 1991; Ullenhag et al., 2006, 2008).

Abagovomab, an anti-idiotype mAb directed against CA125, has shown to induce active immune response against this tumor-associated antigen in a Phase Ib/II clinical trial in advanced ovarian cancer patients. In this study, those patients who developed anti-anti-idiotype (Ab3) response had a significant longer overall survival (Reinartz et al., 2004). On the basis of these encouraging results, a Phase II/III pivotal study (MIMOSA study) to evaluate abagovomab as a maintenance therapy in ovarian cancer patients with no residual disease after frontline therapy was conducted (Pfisterer et al., 2011a). In this study was enrolled 888 patients, and 593 women were treated with abagovomab and 295 women were assigned to treatment in the placebo arm. Unfortunately, the results of this study showed that abagovomab maintenance treatment after debulking surgery and successful platinum and taxane first-line chemotherapy did not prolong progression-free survival in advanced ovarian cancer patients (Pfisterer et al., 2011b).

Also, anti-idiotype mAbs have been used to mimic non-protein antigens in patients with different tumors. One example of these kind of antigens are the gangliosides which are sialic acid-containing glycosphingolipids normally expressed in the membrane of eukaryotic cells (Wiegandt, 1985), but their expression patterns change during oncogenic transformation (Hakomori, 1985; Irie and Ravindranath, 1990).

The anti-idiotype mAb 1A7, which functional mimics the ganglioside GD2, (Sen et al., 1998) has been used as an anti-idiotype vaccine (TriGem) in patients with advanced melanoma (Foon et al., 1998, 2000). The immunization with this anti-idiotype vaccine elicited a strong anti-GD2 antibody response that specifically reacted with tumor cells expressing GD2. Although the studies included a small number of patients, the results suggested that TriGem vaccine may have a role in preventing disease progression and in increasing survival time for patients with advanced malignant melanoma (Foon et al., 2000).

Another antibody used as an anti-idiotype vaccine in cancer patients is the mAb Bec2, which mimics GD3 ganglioside (Chapman and Houghton, 1991). This antibody was studied in melanoma and small cell lung cancer (SCLC) patients, where it induced specific anti-GD3 antibodies, but only in a low percentage of patients (McCaffery et al., 1996; Grant et al., 1999). Furthermore these antibodies were hardly detectable by TLC immunostaining or flow cytometry (Ritter et al., 1991). Despite the low frequency of anti-GD3 antibodies induction in the first trials, a Phase III clinical trial with 515 SCLC patients with a major response to chemotherapy and chest radiation was performed with this antibody. Only one third of the patients elicited an anti-GD3 response. Although this trial failed to show any survival advantage for vaccinated patients, a trend toward prolonged survival was observed in those patients who developed the humoral response (Giaccone et al., 2005).

## NeuGc-CONTAINING GANGLIOSIDES ARE ATTRACTIVE TARGETS FOR IMMUNOTHERAPY OF TUMORS

In particular the Neu-glycolyl (NeuGc)-neuraminic acid variant of gangliosides is widely expressed in most mammalian tissues, but is rarely found in normal human cells. The lack of expression of this type of sialic acid in humans is due to the inactivating mutation in the CMP-Neu5Ac hydroxylase gene, enzyme responsible for Neu5Gc biosynthesis of Neu5Gc-containing molecules (Chou et al., 1998; Irie and Suzuki, 1998; Olson and Varki, 2003). However, the presence of NeuGc-neuraminic acid residues has been reported in different human tumors, detected by antibodies and by chemical analysis (Higashi et al., 1984, 1988; Hirabagashi et al., 1987; Miyake et al., 1990; Devine et al., 1991; Marquina et al., 1996; Malykh et al., 2001; van Cruijsen et al., 2009; Scursoni et al., 2010), where they are known to be immunogenic (Nguyen et al., 2005). Additionally, recent experimental data suggest that NeuGcGM3 ganglioside is relevant for tumor progression (de León et al., 2006).

The most accepted theory for this phenomenon is the incorporation of Neu5Gc from dietary sources. Free sialic acids from the medium can be taken up into cells via pinocytosis. The content of the resulting pinocytotic vesicles and endosomes would eventually be delivered to the lysosome, where a sialic acid transporter then delivers the molecules into the cytosol (Bardor et al., 2005). The explanation for the differential expression of these antigens in human normal and tumor tissues is that the rapidly growing tumor tissues might be more efficient at scavenging Neu5Gc from dietary sources. Furthermore, it has been proposed that the preferential expression of NeuGc in cancers is closely associated with tumor hypoxia. Hypoxic culture of tumor cells induces expression of a sialic acid transporter, sialin, and enhances the incorporation of non-human sialic acid from the external milieu (Yin et al., 2006).

## RACOTUMOMAB ANTI-IDIOTYPE ANTIBODY

We first generated a mAb, named P3, which distinguish between NeuGc and NeuAc gangliosides (Vázquez et al., 1995; Moreno et al., 1998). This mAb also reacted with human melanomas, breast and lung tumors (Vázquez et al., 1995; Alfonso et al., 2002, Alfonso et al., 2007; Neninger et al., 2007). Then, by immunizing Balb/c mice with P3 mAb we obtained an anti-idiotype mAb, named first 1E10 and now racotumomab. This IgG1 Ab2 mAb, was able to block the binding of the Ab1 mAb to NeuGcGM3 (Vázquez et al., 1998).

Our hypothesis was that racotumomab could act as a surrogate of NeuGc-gangliosides generating autologous antibodies in the immunized species which react with the Ab2 idiotype and with the NeuGc-containing gangliosides.

## PRECLINICAL DATA

We tested the hypothesis using different animal models with a different NeuGc-sialic acid expression: mice and monkeys that express NeuGc-glycoconjugates (Chou et al., 1998; Hayakawa et al., 2001) and chickens, which do not express NeuGc-glyco-conjugates in their normal tissues (Fujii et al., 1982; Ledeen and Yu, 1982).

Animals received different doses of preparations containing racotumomab and when we evaluated the serological antibody response we found that all animals of the different species developed Ab3 response. It is noteworthy that in monkeys and chickens where racotumomab is a xenogenic immunoglobulin the serological response against racotumomab was stronger when compared with other isotype-matched mAb used as a control (immunodominance of the idiotype determinants). Besides, the Ab3 antibodies generated by immunization with racotumomab mAb were characterized to share idiotopes with P3 mAb (Ab1), as evidenced by their capacity of inhibiting racotumomab binding to P3 mAb (Ab3 Id+; Vázquez et al., 1998; Hernández et al., 2005).

In contrast, when the antibody response against NeuGc-containing gangliosides induced by racotumomab immunization was studied, a completely different pattern of response was detected. In mice and monkeys, no specific humoral response against NeuGc-containing gangliosides was detected in the sera of racotumomab immunized animals. In the case of mice and monkeys, since NeuGc-containing gangliosides are normal tissue components (Ledeen and Yu, 1982), tolerance mechanisms could be avoiding the immune response against these antigens. These mechanisms are still unknown, but as it has been demonstrated for other self-antigen models (Fillatreau et al., 2002; Takahashi and Sakaguchi, 2003), one possibility is the existence of T and/or B regulatory cells that would modulate the antibody response against NeuGc-containing gangliosides.

On the other hand, most chickens in our study developed a specific Ab3 response against NeuGcGM3 and NeuGcGM2 gangliosides (Ab3 Ag+), due to the immunization with racotumomab (Hernández et al., 2005). In addition, Ag+Id-antibodies were produced in chickens immunized with racotumomab. This was demonstrated by the reactivity of hyperimmune sera to NeuGcGM3 ganglioside after the adsorption of hyperimmune chickens sera with racotumomab, which abrogate the antibody response against this Ab2 (Hernández et al., 2005). The mechanism responsible for the generation of these antibodies is still unknown. A natural immune network could be involved in the generation of this unusual antibody “parallel set”.

Although these results, it is known that the induction of antigen-specific humoral immune response is not predictive of the biological effect induced by an anti-idiotype mAb. Thus, we studied the effect of different preparations containing racotumomab in different experimental tumor murine models.

In BALB/c mice, vaccination with several intraperitoneal doses at 14-day intervals of racotumomab coupled to keyhole limpet hemocyanin in Freund’s adjuvant, significantly reduced subcutaneous tumor growth of F3II carcinoma cells and the formation of spontaneous lung metastases. The effect of racotumomab as a biological response modifier on lung colonization was evaluated in C57BL/6 mice injected with B16 melanoma cells. Interestingly, intravenous administration of uncoupled racotumomab antibody, 10–14 days after inoculation of tumor cells, dramatically reduced the number of experimental metastases in comparison with lungs from mice treated with an irrelevant IgG (Vázquez et al., 2000).

Later, we evaluated the anti-metastatic effect of racotumomab in Alum adjuvant (racotumomab-Alum) in the 3LL-D122 Lewis lung carcinoma model. Racotumomab-Alum immunization was tested in two different settings distinguished by the frequency of the immunizations, the amount of vaccine and the initiation of the vaccine schedule related to the tumor challenge. Independently to the immunization schedule, racotumomab-Alum promoted a significant reduction of spontaneous lung metastases. The therapeutic effect was associated to the increment in the number of T cells infiltrating metastases, a reduction of new blood vessels formation, and the increment of apoptotic tumor cells in lung nodules. It is noteworthy that active immunization with the mAb in Alum does not induce measurable antibodies to racotumomab molecule, the NeuGcGM3 or tumor cells, which may suggest a different mechanism which has to be elucidated (Diaz et al., 2009).

More recently, preclinical studies were carried out with the main objective to determine the anti-tumor effect of racotumo-mab-Alum vaccine coadministered with low dose of cyclophosphamide in a murine mammary carcinoma model, based on their potentially shared antiangiogenic properties and/or a complementary proapoptotic effect by racotumomab-Alum vaccine. The results of the study showed that the combination enhanced the efficacy of chemotherapy or immunotherapy alone in the F3II carcinoma model, both when the mAb was obtained from mice ascites fluid or from bioreactor supernatant (Fuentes et al., 2010; Machado et al., 2011).

## CLINICAL DATA

Taking in account the preclinical results together with the results of the toxicology studies, and after the approval of the Ethical Committees of the hospitals and the Regulatory agencies, different Phase I clinical trials were performed in advanced melanoma (Alfonso et al., 2002), breast cancer (Díaz et al., 2003; Guthmann et al., 2006; Soriano et al., 2011), and SCLC and non-SCLC (NSCLC; Alfonso et al., 2007; Neninger et al., 2007), with the main goals of evaluate the safety and the immunogenicity of racotumomab vaccine. All the patients had previously received the oncospecific treatment established in the Oncological Therapeutic Standards. In most of these clinical trials the patients were injected intradermally with 10 doses of 1 or 2 mg of racotumomab-Alum as base treatment: the first four or five doses at 2-week intervals and the remaining six every 28 days. Reimmunizations were administered if the patients had a favorable clinical status. The results of these Phase I clinical trials evidenced that racotumomab vaccine was well tolerated. Toxicity consisted mainly of local reaction at the injection site with erythema and induration that disappeared in a few days (adverse events grade I/II according to NCI-CTC criteria).

Racotumomab-Alum vaccine resulted very immunogenic, a strong, and specific response against NeuGc-containing gangliosides was detected in the sera of most of the immunized patients by ELISA and TLC-immunostaining (Alfonso et al., 2002; Díaz et al., 2003; Guthmann et al., 2006; Neninger et al., 2007). One interesting finding was the detection of a high level of binding of patient’s hyperimmune sera to NeuGcGM3 ganglioside, after the complete abrogation of the reactivity against racotumomab mAb by adsorbing the patient sera with this Ab2 (Hernández et al., 2008). Also, antibodies able to react with lung carcinoma tissue sections were detected in the sera of immunized lung cancer patients (Alfonso et al., 2007; Neninger et al., 2007).

The finding that in most of the patients treated with racot-umomab-Alum vaccine we detected a relatively high titer of anti-NeuGcGM3 antibodies of both IgM and IgG isotypes is a relevant result taking into account that it is difficult to obtain an IgG antibody response against these antigens (Livingston, 1995). Even the use of anti-idiotype antibodies as protein mimicries of gangliosides does not guarantee the induction of this kind of response. Previously, it was reported that most of the melanoma patients immunized with 1A7 mAb able to mimic GD2 ganglioside developed specific IgG antibodies against this ganglioside (Foon et al., 2000). In contrast, when melanoma and SCLC patients were treated with the anti-idiotype Bec-2 mAb, the percentage of patients that developed anti-GD3-specific antibody response was low, mainly of IgM isotype. The presence of these antibodies was detected by ELISA, but could not be confirmed by TLC immunostaining or flow cytometry (Ritter et al., 1991; McCaffery et al., 1996; Grant et al., 1999). The differential induction of antibody responses against gangliosides could be dependent on their different expression in normal tissues. In fact, studies previously reported showed the relation between the level of ganglioside expression in human and murine normal tissues and their immunogenicity (Chen et al., 2000; Lunn et al., 2000; Bowes et al., 2002). The strong antibody response against NeuGcGM3 induced in patients by the racotumomab-Alum vaccine can be explained because NeuGc-containing gangliosides are not self-antigens in humans (Chou et al., 1998; Irie and Suzuki, 1998; Olson and Varki, 2003).

Although these studies were not designed to evaluate the therapeutic efficacy of the vaccine, a prolonged survival was observed in several patients. In particular, the clinical study performed in NSCLC patients stage IIIb/IV showed encouraging clinical benefit. This clinical trial study included 34 stage IIIb and 37 stage IV NSCLC patients. These patients were treated with the anti-idiotype vaccine, after received standard chemotherapy and radiotherapy, in a compassionate-use basis study. Patients received five bi-weekly injections of 1 mg of racotumomab-Alum, other 10 doses at 28-day intervals and later the patients who maintained a good performance status continued to be immunized at this same time interval. No evidence of unexpected or serious adverse effects was reported, although the patients were repeatedly injected with racotumomab-Alum and even when some of them received more than 15 doses of this vaccine. The overall survival of the patients who entered the study was superior to the one reported (González et al., 2007) for a group of more than one hundred advanced NSCLC patients receiving standard oncospecific treatment in our country (9.93 vs. 4.53 months). In this study, the survival of the patients who started racotumomab-Alum with at least disease stabilization after the end of standard therapy and with a PS1, was significant greater (11.50 months) compared with those patients with progressive disease and/or a PS2 (6.50 months). To assess whether this advantage in survival could be due to the vaccination with racotumomab, a comparison was performed with the median survival time calculated for a subgroup of the control patients with a PS1 who achieved partial response or stable disease after the oncospecific therapy, and there was a statistical significant difference between the two groups (11.50 vs. 5.70 months). Thus, the treatment with the racotumomab-Alum vaccine seems to prolong survival of this group of patients.

Immunological studies performed in 20 of these patients suggested an association between the induction of antibodies against NeuGcGM3 and longer survival times (Hernández et al., 2008). Then, one question we wanted to address was if the generation of NeuGcGM3 specific antibodies in patients could have some biological effect in tumor cells expressing this ganglioside.

Our results from flow cytometry studies, where the sera of patients were incubated with a cell line expressing NeuGcGM3 and the effect on their viability was studied using the propidium-iodine exclusion assay, showed that sera of most of the patients not only recognize the cells, but also directly killed the cells, by a mechanism independent of complement activation. When the cells were examined by scanning electron microscopy it was shown that the cytotoxic hyperimmune patient’s sera produce large lesions on cell’s membranes (Hernández et al., 2011). These results contributed to reinforce the therapeutic potential of anti-idiotype vaccines and the importance of NeuGcGM3 as tumor target.

Then, a Phase II/III randomized, multicentric, double blind, clinical study was performed in advanced NSCLC patients looking to the proof of concept with significant clinical benefit in the patients who were treated with the anti-idiotype vaccine in comparison with the placebo group (manuscript in preparation). Now, a Phase III multinational clinical trial is ongoing to confirm our clinical results in advanced NSCLC patients.

## CONCLUDING REMARKS

Racotumomab-Alum vaccination has proved to be safe and highly immunogenic in patients with advanced melanoma, breast and lung cancer. Racotumomab-Alum vaccination seems to be effective to prolong overall survival in patients with NSCLC and preliminary data suggested a correlation between the induction of antibodies against NeuGcGM3 and increased survival times of vaccinated patients. The current ongoing Phase III clinical trial will give a definitive answer to the potential clinical benefits offered by this vaccine. Furthermore, studies will be required to determine the more efficient combination with chemotherapy or other immune interventions to prevail over the tumor-induced immunosuppression.

## Conflict of Interest Statement

Dr Ana M. Vázquez is an inventor of patents related with P3 mAb and its anti-idiotypes, however, she has signed the assignment of her rights to the assignee Center ofMolecular Immunology. Dr Roberto E. Gómez is a full time employee at ELEA Laboratories.
